# YB‐1 orchestrates onset and resolution of renal inflammation *via IL10* gene regulation

**DOI:** 10.1111/jcmm.13260

**Published:** 2017-06-30

**Authors:** Jialin Wang, Sonja Djudjaj, Lydia Gibbert, Vera Lennartz, Daniel M. Breitkopf, Thomas Rauen, Daniela Hermert, Ina V. Martin, Peter Boor, Gerald S. Braun, Jürgen Floege, Tammo Ostendorf, Ute Raffetseder

**Affiliations:** ^1^ Department of Nephrology and Clinical Immunology University Hospital RWTH‐Aachen Aachen Germany; ^2^ Institute of Pathology University Hospital RWTH‐Aachen Aachen Germany

**Keywords:** renal inflammation, renal fibrosis, YB‐1, IL‐10, fourth intron, ischaemia–reperfusion, LPS

## Abstract

The Y‐box‐binding protein (YB)‐1 plays a non‐redundant role in both systemic and local inflammatory response. We analysed YB‐1‐mediated expression of the immune regulatory cytokine IL‐10 in both LPS and sterile inflammation induced by unilateral renal ischaemia–reperfusion (I/R) and found an important role of YB‐1 not only in the onset but also in the resolution of inflammation in kidneys. Within a decisive *cis*‐regulatory region of the *IL10* gene locus, the fourth intron*,* we identified and characterized an operative YB‐1 binding site *via* gel shift experiments and reporter assays in immune and different renal cells. *In vivo*, YB‐1 phosphorylated at serine 102 localized to the fourth intron, which was paralleled by enhanced IL‐10 mRNA expression in mice following LPS challenge and in I/R. Mice with half‐maximal expression of YB‐1 (*Yb1* ^*+/−*^) had diminished IL‐10 expression upon LPS challenge. In I/R, *Yb1* ^*+/−*^ mice exhibited ameliorated kidney injury/inflammation in the early‐phase (days 1 and 5), however showed aggravated long‐term damage (day 21) with increased expression of IL‐10 and other known mediators of renal injury and inflammation.

In conclusion, these data support the notion that there are context‐specific decisions concerning YB‐1 function and that a fine‐tuning of YB‐1, for example, *via* a post‐translational modification regulates its activity and/or localization that is crucial for systemic processes such as inflammation.

## Introduction

The YB‐1 is a transcriptional and translational factor, which regulates many cellular processes such as cell proliferation, DNA repair, cellular stress response, cell differentiation, embryonic development and inflammation 1, 2, 3. In both, systemic and localized inflammatory responses to infectious stimuli 4 as well as during ‘sterile’ inflammation 3, 4, YB‐1 regulates expression of pro‐inflammatory mediators. In the onset of inflammation, YB‐1 up‐regulates the expression of pro‐inflammatory factors such as interleukin (IL)‐6 5 and CCL5 4, 6, 7, 8. CCL5 is an important inducer of immune cell infiltration, which is transcriptionally and translationally 4 regulated by YB‐1 in monocytes/macrophages 8 as well as in human arterial smooth muscle cells 7. However, anti‐inflammatory properties of YB‐1 *via* a trans‐repressive capacity on the *Ccl5* promoter upon macrophage differentiation are also described 8. Thus, YB‐1 influences the early‐phase of inflammation and also seems to contribute to its termination in the later phase. However, so far, it is unknown whether this dual role of YB‐1 also encompasses the regulation of proteins with anti‐inflammatory properties such as IL‐10.

Experimental renal ischaemia–reperfusion (I/R) allows to analyse the onset of acute kidney injury (AKI) and late sequelae including renal regenerative processes 9, 10. Following lack of blood supply with hypoxia (ischaemia) and restoration of the circulation (reperfusion), reversible renal inflammation occurs. Experimental I/R models one of the leading cause of AKI in both native and transplant kidneys 11, 12, which is still associated with high mortality and morbidity 13, 14. In I/R‐induced AKI, an immune response, is initiated following the ischaemic insult *via* the extravasation of immune cells through the disrupted endothelium. Early inflammatory events occur in the microvascular and tubulointerstitial compartment of the medulla with leukocytes and platelets blocking renal microcirculation. Subsequently, this strong inflammatory response further spreads to the renal cortex and chemokine expression is still high during the repair phase 10.

Both, the early‐ and the late‐phase of kidney I/R are characterized by infiltration of immune cells including different subtypes of T lymphocytes 15, macrophages and dendritic cells 16 that can facilitate injury but also promote repair.

Immune regulatory mediators, such as IL‐10, have been described as renoprotective, as they alleviate kidney damage in nephritis 17, 18. IL‐10 suppresses activation and accumulation of innate immune cells. Recently, regulatory T cell (Treg)‐derived IL‐10 has been described to exert an important protective role in murine models of I/R 17 and crescentic glomerulonephritis 18. Beyond immune cells, resident renal cells, that is mesangial 19 and tubular 20 cells, are also able to produce IL‐10.

In this study, we unravel molecular mechanisms underlying YB‐1‐induced IL‐10 production during immune responses to microbial PAMPs as well as during ‘sterile’ inflammation and clarify the general capacity of YB‐1 to mediate pro‐ and anti‐inflammatory processes in these models in wild‐type (WT) and *Yb1* ^*+/−*^ mice. As it controls IL‐10 expression, we demonstrate that YB‐1 orchestrates both onset and resolution of the inflammatory responses.

## Materials and methods

### Cell culture

Rat mesangial cells (rMCs) and human T lymphocytes (HUT78T) were cultured in RPMI 1640 medium, and human tubular epithelial cells (HK‐2) were incubated in low‐glucose DMEM medium with 1% MEM‐NEAA and 10 μg/ml insulin. All media were supplemented with 10% FCS, 100 units/ml penicillin and 100 units/ml streptomycin. All cell lines were maintained at 37°C in humidified air with 5% CO_2_. Some cells were incubated with ochratoxin A (OTA, Santa Cruz Biotechnology, Heidelberg, Germany, 20 μM) for the indicated times or with 10 ng/ml LPS (Sigma‐Aldrich, Steinheim, Germany) or with PBS alone for 6 hrs. To prevent phosphorylation of YB‐1, cells were pre‐incubated with 10 μM Ly294002 (Calbiochem, Darmstadt, Germany) for 2 hrs. Results were confirmed in at least three independent experiments.

### Plasmids

Plasmids encoding the luciferase gene solely or the luciferase gene fused with the *IL10* fourth intron (630 bp) were described before 21. A full‐length YB‐1 expression plasmid (YB‐1‐pSG5) was kindly donated by J.Ting (Lineberger Comprehensive Center, University of North Carolina).

### Transient transfection

Transient transfections in HK‐2 cells were performed using calcium phosphate precipitates as described previously 22. rMCs (2 × 10^5^ cells/well) were transiently transfected with the lipid‐based transfection reagent *FuGENE 6 HD* (Promega, Madison, WI, USA) according to the manufacturer's instruction. Human HUT78T cells were transfected by means of electroporation; 2.5 × 10^6^ cells resuspended in 1 ml of RPMI 1640 medium supplemented with 20% FCS were added to electroporation cuvettes (0.4 cm gap, Bio‐Rad, Hercules, CA, USA) together with a total amount of 20 μg of plasmid DNA, respectively. The mixture was incubated for 5 min. on ice, and cells were electroporated at 250 V/1100 μF in a Gene Pulser II electroporation system (Bio‐Rad). Cells were incubated another 5 min. on ice prior to resuspension in 2 ml of RPMI 1640 medium supplemented with 20% FCS, transferred to six‐well tissue culture plates and incubated at 37°C and 5% CO_2_.

### Luciferase reporter assay

Luciferase reporter constructs were introduced into HUT78T, HK‐2 cells and rMCs by the indicated transfection protocols mentioned above. Luciferase assay using the Dual‐Luciferase^®^ Assay System (Promega) was performed according to the manufacturer's instruction and as described before 6. Results were confirmed in at least three independent experiments.

### Animal experiments

The local review board approved all animal studies according to prevailing guidelines for scientific animal experimentation. Animals were held in cages with constant temperature and humidity with drinking water and food *ad libitum*. Mice heterozygously targeted for a disruption of the *Yb1* locus (*Yb1* ^*+/−*^, C57BL/6 background) were kindly donated by Timothy J. Ley (Section of Stem Cell Biology, Division of Oncology, Washington University Medical School, St. Louis, MO). Homozygous YB‐1‐deficient mice are not viable and die before birth due to neuronal disorders, which precludes their use 23.

#### Ischaemia/Reperfusion (I/R)‐induced inflammation model

For the unilateral renal I/R‐induced inflammation model, 13‐ to 16‐week‐old male *Yb1* ^*+/−*^ mice and their age‐matched WT littermates (*Yb1* ^*+/+*^, WT) were anaesthetized with *i.p*. injection of 0.01% xylazine hydrochloride and 1% ketamine hydrochloride mixture diluted in 0.9% NaCl acclimatized on a warmed electrical plate for 15 min. (constant plate temperature of 37 °C). The abdominal cavity was exposed through left lateral incision, and the left renal pedicle was carefully isolated. The pedicle occlusion was performed using non‐traumatic vascular clamps for 30 min. or 25 min., and the efficacy of occlusion was confirmed by colour changing in the entire kidney. Sham‐operated mice underwent the same procedure without clamping the vessels. During the whole procedure, mice were maintained at 37°C using a warmed electrical plate. Mice were killed by cervical dislocation on days 1, 5 and 21 after renal reperfusion. The left kidney tissues were taken for immunohistochemical analyses, protein and mRNA extraction.

#### LPS (Lipopolysaccharide)‐induced inflammation model

For the LPS‐induced inflammation model, 18‐ to 22‐week‐old *Yb1* ^*+/−*^ mice and their age‐matched WT littermates received a single dose of LPS (1.5 mg/kg body weight, 6 and 12 hrs) dissolved in PBS or PBS alone as control by *i.p*. injection as described before 4.

### Quantitative real‐time RT‐PCR

For quantitative real‐time RT‐PCR, total RNA was purified from cortical kidney tissue by TRIzol reagent (Life Technologies, Darmstadt, Germany), according to the manufacturer's protocol. First‐strand cDNA was synthesized with Moloney murine leukaemia virus reverse transcriptase (Invitrogen, Carlsbad, CA). qRT‐PCR was carried out with the 7300 real‐time PCR system (Applied Biosystems, Darmstadt, Germany). Taqman master mix and Taqman primer sets were obtained for mouse *Il10* (Mm00439614_m1), mouse chemokine *Ccl5* (Mm01302428_m1), rat *Il10* (Rn00563409_m1), human *IL10* (Hs00961622_m1), human *YB1* (Hs02742754_g1) and eukaryotic 18S rRNA (Hs99999901_s1) as an internal control from Applied Biosystems.

For *Ngal* detection, 0.75 μl cDNA and 12.5 μl PCR Master Mix (qPCR Core kit with SYBR Green I; Eurogentec, Seraing, Belgium) were used for each reaction in a total of 25 μl volume. *Gapdh* cDNA amplification was used as an internal standard. The primer sequences for *Ngal* and *Gapdh* were as follows: *Gapdh*, 5'‐GGCAAATTCAACGGCACAGT‐3' and 5'‐GATGGTGATGGGCTTCCC‐3'; *Ngal*, 5''‐GGCCTCAAGGACGACAACA‐3' and 5'‐TCACCACCCATTCAGTTGTCA‐3'. Levels of gene expression were determined by the comparative deltaCT methodology.

For ChIP analyses, real‐time qPCR was performed using a qPCR Core Kit for SYBR Green I (Eurogentec). The conditions for all Taqman PCRs were 50°C for 2 min., followed by 40 cycles of 95°C for 15 sec. and 60°C for 1 min.

### Chromatin immunoprecipitations (ChIPs)

ChIP assay was performed using SimpleChip Plus Enzymatic Chromatin IP Kit (Cell Signaling, Danvers, MA, USA) according to the manufacturer's protocol and as described before 24. Antibodies specific to histone H3 (Cell Signaling) as a positive control, YB‐1^CSD^ (Eurogentec) and p‐YB‐1^S102^ (Cell Signaling), were added for immunoprecipitation and compared to control non‐specific IgG (Cell Signaling). A proportion (20%) of the samples was kept as ‘Input’ to represent the PCR amplification of the total sample.

The real‐time qPCR primer sequences used to amplify the regions within the murine *Il10* fourth intron were as follows: 5'‐TGTGGGAACCCAGCAAATG‐3'; 5'‐TGTAACTGAGGTGGTGGCTTTCTA‐3'. The amount of immunoprecipitated DNA was subtracted by the amplified DNA that was bound by the non‐specific IgG antibody and calculated relative to the respective input DNA. Further, real‐time qPCR products were separated on a 3% agarose gel containing 0.01% GelRed (Biotium, Hayward, CA).

### Electrophoretic mobility shift analyses

The biotinylation of synthetic DNA probes corresponding to the antisense strands of the human *IL10* fourth intron sequences was performed with the *Biotin 3' End DNA Labeling Kit* (Thermo Fisher Scientific, Rockford, Illinois, USA) according to the manufacturer's instructions. Nuclear cell extract preparation and EMSA with Light‐Shift chemiluminescent EMSA kit (Thermo Fisher Scientific, Rockford, Illinois, USA) were performed as described before 6.

For supershift analyses, the following antibodies were incubated with nuclear proteins 20 min. prior to addition of probes: IgG as a negative control (Millipore, Temecula, CA, USA), C‐terminal YB‐1 (Sigma‐Aldrich)‐ and YB‐1^CSD^ (Eurogentec)‐specific antibodies. Supershifted complexes were indirectly seen from decreased band intensities on the blot, whereas bands should not change when control non‐specific IgG was added to the binding reactions.

### Cortical kidney lysates and Western blot analysis

Cortical kidney lysates were prepared as described before 4. Protein concentrations were determined using BC Assay Protein Quantitation Kit (Uptima Interchim, Montluçon, France) according to the manufacturer's protocol; 10 to 30 μg protein of kidney extracts was subjected to SDS‐PAGE. Proteins were subsequently transferred to nitrocellulose membranes and visualized by Lumi‐Light and Lumi‐Light^PLUS^ Western blotting substrate (Roche Diagnostics GmbH, Mannheim, Germany), respectively. The primary antibodies anti‐YB‐1 against the protein C‐terminus (Sigma‐Aldrich) and anti‐p‐YB‐1 against phosphorylated Ser‐102 (Cell Signaling) and anti‐mouse NGAL (R&D systems, USA) were used. To ensure equal protein loading, the blots were additionally incubated with a monoclonal GAPDH‐specific antibody (Novus Biologicals, Littleton, CO).

### Immunohistochemistry/immunofluorescence

Immunohistochemistry was performed in 1‐μm‐thick paraffin sections of methyl Carnoy's‐fixed specimens using the Vectastain Avidin/Biotin System (Vector Laboratories, Burlingame, CA), as described before 25. Renal tissues were stained using the following primary antibodies: anti‐human Col1A1 (Southern Biotech, Birmingham, AL), anti‐Kim1 (R&D systems, USA), anti‐mouse F4/80 (Serotec, Düsseldorf, Germany), anti‐proliferating cell nuclear antigen (PCNA) (Leinco Technologies, St. Louis, MO) and anti‐Ly6G (BD Biosciences, San Jose, CA). Negative controls for the immunohistochemical procedures consisted of substitution of the primary antibody with non‐immune IgG. Quantification of Ly6G was described before 4. Total number of PCNA‐stained nuclei in renal tubuli was counted in 25 randomly selected fields at ×200 magnification. For staining quantification of collagen and F4/80, 20 consecutive images of renal cortex were taken per section. The percentage of the positively stained area was extracted for intensity using ImageJ software (Wayne Rasband, NIH) and a mean area was calculated. Periodic acid–Schiff (PAS) staining was performed as described before 25, and the tubular injury was scored on a scale of 0–4: 0 = none; 1 = 0–25%; 2 = 25–50%; 3 = 50–75%; 4 = more than 75%. The total score is the calculated average of all tubular scores.

### Statistical analysis

Values are expressed as means ± S.D. Statistical significance was evaluated using the Student's *t*‐test or ANOVA and Bonferroni *post hoc* test, whenever more than two groups were compared, with significance accepted when *P* value was less than 0.05. All *in vitro* experiments were performed at least in triplicate.

## Results

### YB‐1 regulates IL10 expression *via* a regulatory element within the *IL10* gene locus

**Figure 1:**
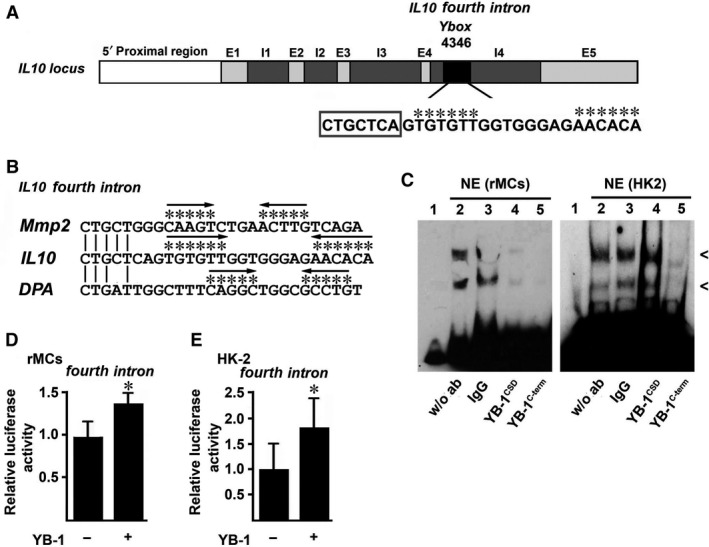
(**A**) Inspection of fourth intron within the human *IL10* gene locus revealed a putative YB‐1‐binding site starting at 4346 bp. (**B**) Comparison with known YB‐1‐binding motifs of the *matrix metalloproteinase2* (*Mmp2*) and *DNA polymerase‐*α (*DPA*) genes revealed strong homologies and an inverted repeat motif (indicated by stars and arrows). (**C**) Nuclear protein extracts from rMCs and HK‐2 cells were prepared, and complex formation with the oligonucleotide that encompasses the *Ybox* within the *IL10* fourth intron was assessed. Two strong high‐mobility nucleoprotein complexes appeared (<) which were strongly weakened especially in the presence of C‐terminal YB‐1‐specific polyclonal antibody but not with non‐specific IgG antibody as control. (**D/E**) Relative *IL10* fourth intron luciferase activity was enhanced after YB‐1 overexpression in rMCs (**D**) and in HK‐2 cells (**E**). Experiments were performed in at least three independent experiments, each performed in triplicate. Data are expressed as mean values ± S.D. *P < 0.05. *NE*, nuclear extract.

To examine the impact of YB‐1 on *IL10* expression, we inspected the fourth intron (4^th^ intron), which constitutes an important intronic enhancer element within the human *IL10* gene locus 21, for potential YB‐1 binding sites. We detected a putative *Y‐box* with an adjacent inverted repeat motif (indicated by asterisks in Fig. 1A) starting at 4346 bp within the *IL10* gene. This element displayed strong homologies with known YB‐1‐binding motifs within the matrix *metalloproteinase2* (*Mmp2)* 26 and *DNA polymerase‐*α (*DPA*) 27 gene loci (Fig. 1B).

Electrophoretic mobility supershift assays (EMSA) were performed with oligonucleotides that encompass the *Y‐box* within the fourth intron of the human *IL10* gene. Indeed, we observed protein–DNA complexes (indicated by arrowheads in Fig. 1C) when nuclear extracts of renal cells, namely rat mesangial cells (rMCs, left) and human tubular cells (HK‐2, right) that are known to produce IL‐10 19, 20, were included. Specifically, addition of an antibody against the C‐terminus of YB‐1 protein confirmed its binding to this site. Consequently, YB‐1 overexpression enhanced the *IL10* fourth intron activity in rMCs (Fig. 1D) and HK‐2 cells (Fig. 1E).

**Figure 2:**
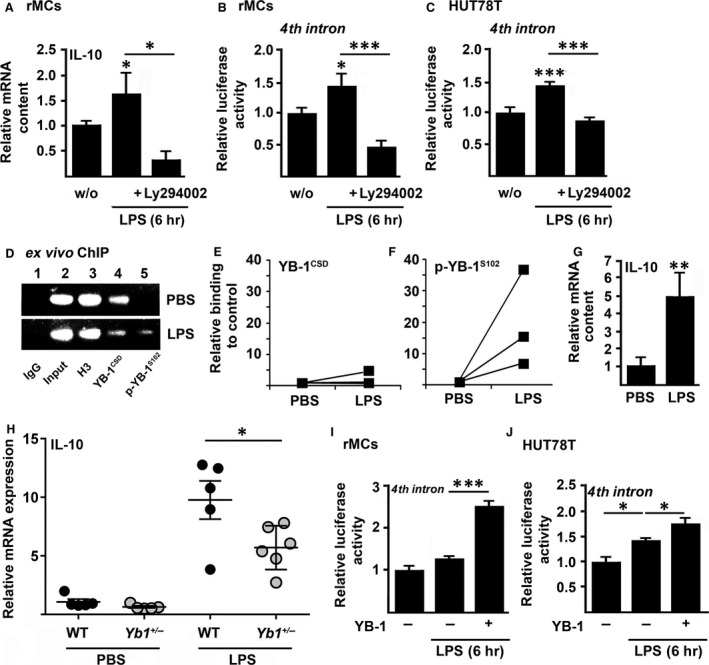
(**A–C**) Relative IL‐10 mRNA expression (**A**) and *IL10* fourth intron luciferase activity **(B/C)** following LPS (10 ng/ml, 6 hr) was decreased through prior incubation with Akt/PKB kinase inhibitor Ly294002 (10 μM) in rMCs (**A/B**) and HUT78T cells **(C)**. (**D–F**) *Ex vivo* ChIP assay revealed binding especially of p‐YB‐1^S102^ to the *Il10* fourth intron in murine kidneys 12 hrs following LPS injection (1.5 mg/kg BW,* i.p*.). Used were anti‐YB‐1‐ (**D/E**) and anti‐p‐YB‐1^S102^‐(**D/F**) specific antibodies or unspecific IgG (negative control) with oligos within *Il10* fourth intron region. Furthermore, amount of included DNA was tested without preceding immunoprecipitation (input). (**G**) Enhanced transcript numbers of IL‐10 in murine kidneys following LPS challenge. (**H**) mRNA expression of IL‐10 was increased in kidneys of both WT and *Yb1* ^*+/−*^ animals 6 hrs after LPS injection (1.5 mg/kg BW,* i.p*.) compared with PBS control animals, but the increase was significantly dampened in *Yb1* ^*+/−*^ mice (*n* = 5–6). (**I/J**) Enhanced YB‐1 expression resulted in elevated *IL10* fourth intron luciferase activity in rMCs (**I**) and in T cells (HUT78) (**J**) following LPS challenge (10 ng/ml, 6 hr). Data are expressed as mean values ± S.D. *P < 0.05; **P < 0.01; ***P < 0.001.

For some genes, Akt‐mediated YB‐1 phosphorylation at serine residue 102 (p‐YB‐1^S102^) 28 is a prerequisite for its *trans*‐activating capacities 6, 29 and previous studies have revealed YB‐1 phosphorylation in LPS‐challenged primary MCs and in a model of acute LPS‐triggered nephritis *in vivo* 4. Thus, we next asked whether targeting Akt/PKB kinase activity also affects *IL10* expression upon exposition to microbial PAMPs. Pre‐incubation with kinase inhibitor Ly294002 resulted in a complete blockade of LPS‐triggered elevated expression of IL‐10 (Fig. 2A) and activation of the fourth intron measured by a reporter assay in rMCs (Fig. 2B). Comparable results were obtained in T lymphocytes (HUT78T) (Fig. 2C).

Next, we addressed the impact of (phospho)‐YB‐1 on renal *Il10* expression in mice. We compared binding of YB‐1 (Fig. 2D and E) and p‐YB‐1^S102^ (Fig. 2D and F) to the *Il10* fourth intron in kidneys of three mice challenged with LPS or with PBS as control. Particularly, binding of p‐YB‐1^S102^ to the fourth intron was enhanced 12 hrs following LPS treatment (Fig. 2F) along with elevated renal IL‐10 mRNA expression (Fig. 2G). The importance of YB‐1 for *Il10* gene expression during LPS‐triggered inflammation was further underlined by a reduced mRNA expression (Fig. 2H) in kidneys of *Yb1* ^*+/−*^ mice that display half‐maximal expression of YB‐1 at both the transcript and protein levels 4, 23, 30 compared to their wild‐type (WT) littermates. *Vice versa*, an increased YB‐1 content enhanced *IL10* fourth intron activity in renal mesangial cells (Fig. 2I) and in T lymphocytes (Fig. 2J) following LPS activation.

Thus, (phospho)‐YB‐1 binds to *IL10* fourth intron and hereby regulates early adjustment of inflammation *via* expression of immune regulatory mediator IL‐10 in renal and immune cells.

### Half‐maximal YB‐1 expression results in decreased tubular damage in the early‐phase of I/R but hinders renal regeneration at later stages

**Figure 3:**
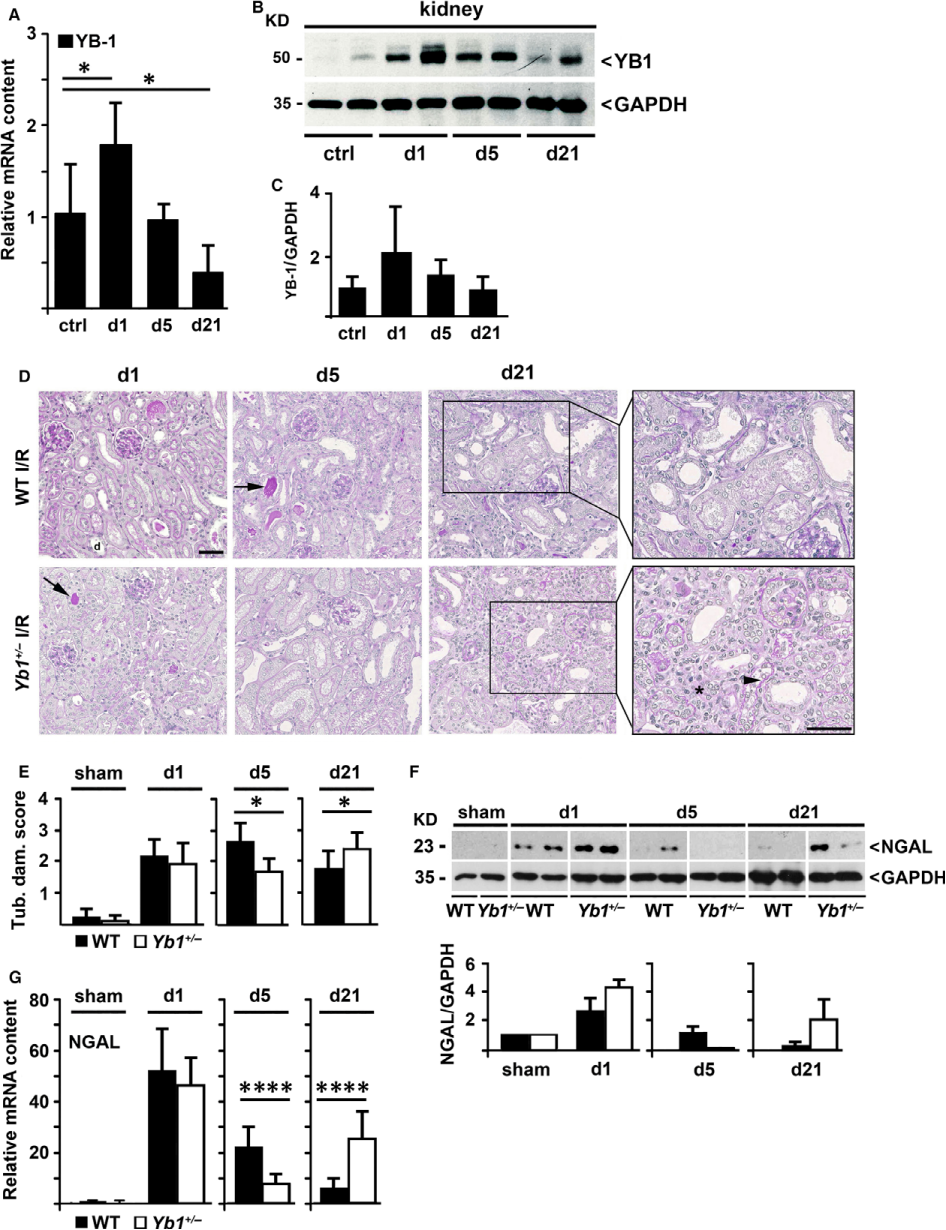
(**A**) Transient upregulation of YB‐1 mRNA expression in the time course of renal I/R model (*n* = 5–10). (**B/C**) Western blot analyses (**B**) of protein lysates for YB‐1 obtained from control (ctrl) and ischaemic kidneys at days 1, 5 and 21 and quantification thereof (**C**). (**D/E**) Tubular damage in PAS‐stained cortical tissue in I/R groups. Representative images (**D**) and quantification (**E**) of renal damage indicated by acute tubular dilatation (d), necrosis (arrowhead) and loss of proximal tubule brush borders revealed less pronounced damage on day 5 in *Yb1* ^*+/−*^ compared to WT I/R mice but more on day 21 (*n* = 5–10). (**F**) Western blot analyses of renal protein extracts of sham, I/R WT and *Yb1* ^*+/−*^ mice with anti‐NGAL Ab and quantification thereof (lower panel). (**G**) Transcript level of NGAL following I/R in WT and *Yb1* ^*+/−*^ mice. Band intensities were quantified by densitometry, and values were normalized against GAPDH. Relative band intensities are depicted in histograms (*n* = 5–8). Scale bars, 50 μm. Data are expressed as mean values ± S.D. *P < 0.05; ****P < 0.0001.

To further investigate the regulatory role of YB‐1 not only in driving acute inflammation but also to monitor progression and resolution of renal inflammation/regeneration, we used a second model of kidney damage associated with sterile inflammation, the unilateral renal ischaemia–reperfusion injury model (I/R) in the early (day 1 after I/R), established (day 5) and advanced disease stage (day 21). In this model, treatment with IL‐10 has been previously shown to ameliorate both renal and systemic inflammation 31. During the time course of I/R, YB‐1 mRNA was significantly up‐regulated in kidney cortex on day 1 (Fig. 3A), returned to normal levels on day 5 and was significantly reduced on day 21. Western blot analyses confirmed transient upregulation of renal YB‐1 (Fig. 3B/C).

In order to elucidate YB‐1's function in this model, we used *Yb1* ^*+/−*^ mice and compared these to WT littermates. Histological examination revealed tubular damage in all I/R groups, indicated by loss of the brush border, cast formation (arrow), acute tubular dilatation (d) and necrosis (arrowhead) (Fig. 3D). Compared to WT animals, *Yb1* ^*+/−*^ mice exhibited less pronounced damage on days 1 and 5. However, 21 days after I/R, mice with half‐maximal YB‐1 expression exhibited significantly stronger damage compared to WT mice, demonstrated by the tubular damage score (Fig. 3D/E) and by areas of exceeding inflammation/fibrosis especially in *Yb1* ^*+/−*^ mice (Fig. 3D, boxed region with a higher power image; marked by asterisk).

**Figure 4:**
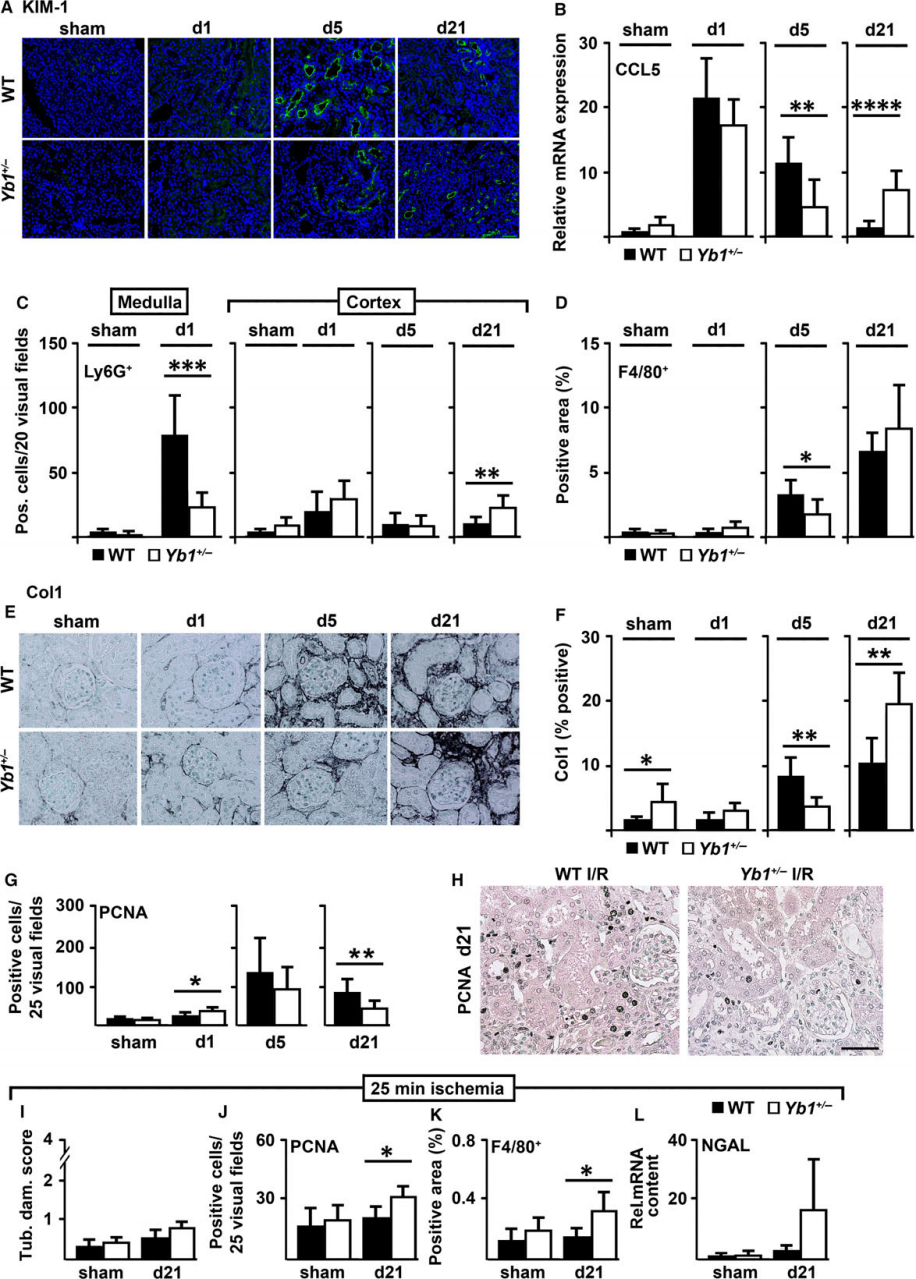
(**A**) Immunofluorescence of tubular damage marker KIM‐1 in WT and in *Yb1* ^*+/−*^ mice. (**B**) Transcript numbers of chemokine CCL5 were determined by qRT‐PCR in kidneys of WT and *Yb1* ^*+/−*^ mice following I/R at different time‐points (*n* = 5–10). (**C/D**) Numbers of renal infiltrating immune cells positive for Ly6G in renal medulla on day 1 and in renal cortex on days 1, 5 and 21 (**C**) and immune cells positive for F4/80 in cortex (**D**) in *Yb1* ^*+/−*^ compared to WT mice following I/R (*n* = 5–14). (**E/F**) Representative images of Col1 immunostaining (**E**) and quantification thereof by computer‐based morphometric analyses of the positively stained cortical area (%) (*n* = 5–7) (**F**). In *Yb1* ^*+/−*^ mice, cortical Col1A1 deposition was significantly less on day 5 but more on day 21 compared to their WT littermates. (**G/H**) PCNA ^+^ tubular nuclei in post‐ischaemic *Yb1* ^*+/−*^ mice in comparison with WT mice on different days (**G**) and representative images on day 21 (*n* = 5–11) (**H**). (**I–L**) Tubular damage in PAS‐stained cortical tissue in I/R groups **(I)** and **(J/K)** quantification of tubular PCNA ^+^ nuclei (**J**) and F4/80^+^ cells (**K**) and NGAL mRNA expression (**L**) in kidneys following shortened ischaemia (25 min.) time (*n* = 4–5). Scale bars, 50 μm. Data are expressed as mean values ± S.D. *P < 0.05; **P < 0.01; ***P < 0.001; ****P < 0.0001.

Our findings were further demonstrated by expression of the tubular damage markers neutrophil gelatinase‐associated lipocalin (NGAL) (Fig. 3F/G). In WT mice, a transiently elevated expression of NGAL protein (Fig. 3F) and NGAL mRNA (Fig. 3G) was observed that peaked on day 1 and continuously decreased in the subsequent observation period until day 21. In contrast, in *Yb1* ^*+/−*^ animals, NGAL mRNA expression significantly decreased on day 5, but significantly increased again on day 21 compared to WT mice (Fig. 3G, white bars). Elevated NGAL protein was detectable on day 21 only in *Yb1* ^*+/−*^ mice (Fig 3F, far right). Augmented tubular damage on day 21 in *Yb1* ^*+/−*^ mice was additionally confirmed by immunofluorescence of kidney injury molecule (KIM)‐1 (Fig. 4A).

Temporal mRNA expression of CCL5, a key chemokine in tissue inflammation, was lower in *Yb1* ^*+/−*^ compared to WT animals at earlier time‐points in I/R, and however was significantly elevated on day 21 (Fig. 4B). This shifted immune response was reinforced by the analysis of infiltration of Ly6G^+^ and F4/80^+^ immune cells. In I/R, early inflammatory events start from the medulla and further spreads to the renal cortex and this is triggered among others by neutrophils. In the medulla of *Yb1* ^*+/−*^ mice, markedly fewer Ly6G^+^ cells were detected on day 1; however at later time‐points, increased numbers of medullary Ly6G^+^ cells were detected in none of the two genotypes (data not shown). However, significant more renal cortical Ly6G^+^ cells were detected on day 21 in *Yb1* ^*+/−*^ compared to WT mice (Fig. 4C). On day 5, the number of cortical F4/80^+^ monocytes/macrophages was significantly higher in cortices of WT compared to *Yb1* ^*+/−*^ mice whereas on day 21 no statistical significant difference was observed between the two genotypes (Fig. 4D).

Following I/R, renal cortical deposition of collagen type 1A (Col1A), a major extracellular matrix (ECM) component in renal fibrotic tissue, was significantly higher in WT compared to *Yb1* ^*+/−*^ animals on day 5, however significantly lower on day 21 (Fig. 4E/F). In line with this, expression of PCNA in tubules as an index of tubular proliferation but also of tubular repair and renal regeneration was significantly higher in *Yb1* ^*+/−*^ than in WT animals on day 1 but lower on day 21 (Fig. 4G/H).

To further explore differences during the regeneration processes in both genotypes, ischaemia time was shortened from 30 min. to 25 min. and expectedly kidneys analysed on day 21 exhibited considerably less tubular damage (Fig. 4I compared to Fig 3E). Advanced regeneration was also reflected in PCNA^+^ tubular nuclei (Fig. 4J) and of F4/80^+^ cells (Fig. 4K) that was already lowered again in WT, however still significantly elevated in *Yb1* ^*+/−*^ mice. Furthermore, expression of NGAL tended to be higher in *Yb1* ^*+/−*^ mice but did not reach significance (Fig. 4L).

Taken together, *Yb1* ^*+/−*^ mice exhibit an ameliorated inflammatory response and kidney damage at the earlier stages following I/R but exhibit aggravated kidney injury, inflammation, fibrosis and less regeneration on day 21.

### YB‐1 modulates renal IL‐10 expression following I/R

**Figure 5:**
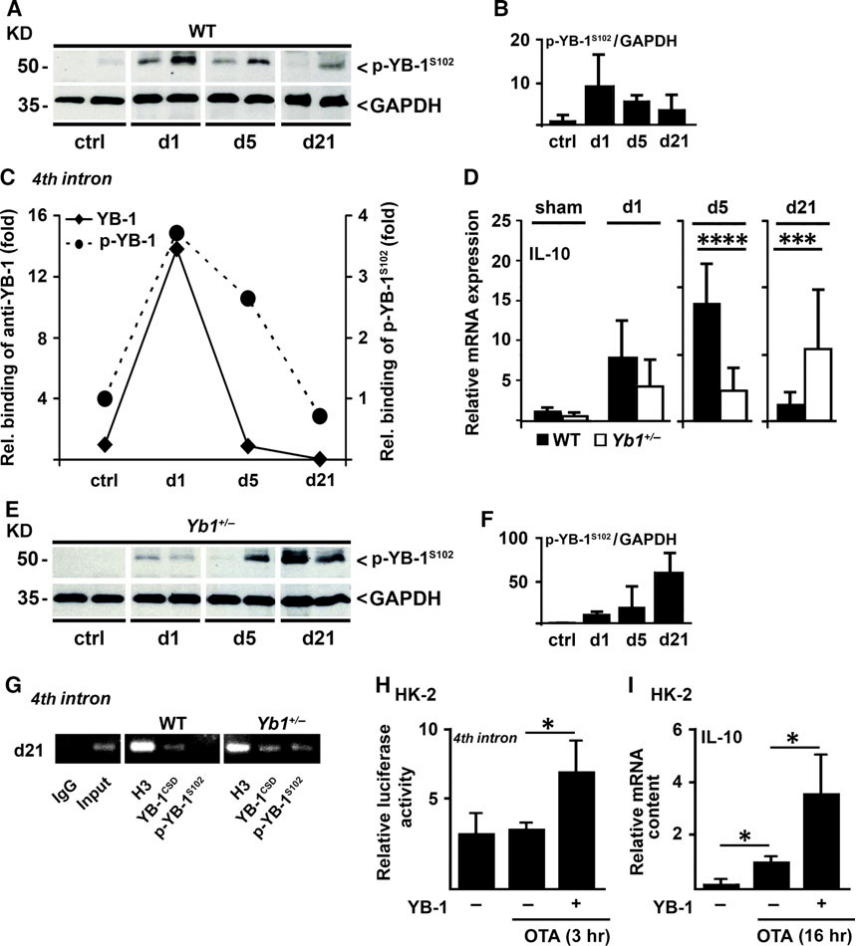
(**A/B**) Western blot analyses of renal protein extracts of WT mice with anti‐p‐YB‐1^S102^ Ab (**A**) and quantification thereof (**B**). (**C**) *Ex vivo* ChIP assay revealed binding of YB‐1 (diamonds) and p‐YB‐1^S102^ (circles) to the *Ybox* within the *Il10* fourth intron in kidneys following I/R that was especially pronounced on day 1 but in case of p‐YB‐1^S102^ still present on day 5. (**D**) IL‐10 mRNA expression in WT (black bars) and *Yb1* ^*+/−*^ (white bars) mice in the time course of I/R (*n* = 5–11). (**E/F**) Western blot analyses of renal protein extracts of *Yb1* ^*+/‐*^ mice with anti‐p‐YB‐1^S102^ Ab (**E**) and quantification thereof (**F**). (**G**) Enhanced binding of p‐YB‐1^S102^ to the *Il10* fourth intron in kidneys following I/R was demonstrated by *ex vivo* ChIP assay. (**H/I**) OTA (20 μM) that induces damage in tubular cells enhanced *IL10* fourth intron luciferase activity (**H**) and IL‐10 mRNA expression (**I**) in renal tubular (HK‐2) cells following YB‐1 overexpression. Band intensities were quantified by densitometry, and values were normalized against GAPDH. Relative band intensities are depicted in histograms. Data are expressed as mean values ± S.D. *P < 0.05; *** *P* < 0.001; ****<0.0001.

To directly address anti‐inflammatory properties of YB‐1 in the different phases after I/R, we monitored presence of p‐YB‐1^S102^ and its binding to the *Il10* fourth intron region on days 1, 5 and 21 by *ex vivo* ChIP analyses of murine kidneys. Analyses of kidney extracts from WT animals exhibited phosphorylation of YB‐1 especially at the two early time‐points following I/R (Fig. 5A/B). On day 1, enhanced binding of p‐YB‐1^S102^ (about fourfold) to the fourth intron of *Il10* was observed that decreased only slightly on day 5 but clearly dropped until day 21 (Fig. 5C, broken line). In contrast to p‐YB‐1^S102^, binding of total YB‐1 to the fourth intron was already strongly diminished on day 5 (Fig. 5C, solid line). During the time course of I/R, a transiently elevated renal expression of IL‐10 mRNA was observed, peaking on day 5 (Fig. 5D, black bars). Thus, at the time of high *Il10* gene activation, the ratio between p‐YB‐1^S102^ and total YB‐1 shifted towards p‐YB‐1^S102^. In *Yb1* ^*+/−*^ compared to WT mice, a strongly and significantly reduced expression of renal IL‐10 mRNA was observed in the acute phase of I/R, especially on day 5 (Fig. 5D, white *versus* black bars). However, on day 21, *Yb1* ^*+/−*^ mice exhibited significantly enhanced IL‐10 expression compared to their WT littermates that was paralleled by highest renal p‐YB‐1^S102^ content on day 21 (Fig. 5E/F) and binding of YB‐1/p‐YB‐1^S102^ to the *Il10* fourth intron (Fig. 5G).

Subsequent studies in damaged proximal tubular HK‐2 cells showed that a higher YB‐1 expression enhanced *IL10* fourth intron activity (Fig. 5H) with a profound increase in IL‐10 mRNA expression (Fig. 5I). Tubular damage was induced by the tubular cell toxin ochratoxin A (OTA) 32.

Taken together, IL‐10 expression is transiently elevated during the time course of I/R and is regulated by binding of p‐YB‐1^S102^ to the fourth intron region within the *Il10* gene. In *Yb1* ^*+/−*^ mice, reduced expression of IL‐10 at early time‐points occurs that might be responsible for the renewed inflammatory response on day 21.

## Discussion

In this study, we identified YB‐1 as an important regulator of the immune regulatory cytokine IL‐10 *via* its fourth intron region in endotoxemia and in I/R‐induced sterile inflammation and demonstrated that YB‐1 exerts both pro‐ and anti‐inflammatory properties in the course of inflammation.

Over the last years, YB‐1 turned out to be an important player in modulating inflammatory events [3, 5, 6, 7, 8, 22, 24, 33, 34]. Transient upregulation of YB‐1 occurs during the acute phase of inflammation in experimental inflammation models in kidneys 4 and in hearts recovering from I/R injury 3, where the expression of YB‐1 in the myocardium was markedly increased in the early‐phase after reperfusion. This is in line with our observation in the renal I/R model, where YB‐1 mRNA and protein expression increased during the onset of inflammation, albeit to a lower extent as compared to other models. Recently, a decrease of YB‐1 protein content 24 hrs after I/R in kidneys was described 35. Conceivably, these opposing observations relate to differences in the experimental setting, for example bilateral 35 *versus* unilateral clamping that can cause different systemic effects with impact on kidney function.

Opposite effects of YB‐1 during inflammatory processes have been described for *CCL5* gene regulation in monocytes and in differentiated macrophages in the course of inflammatory responses 6, 7, 8 and also for IL‐6 generation following LPS challenge. In the latter, YB‐1 acts by differential control of IL‐6 mRNA levels as an inductor in dendritic cells and as a repressor in infiltrating macrophages 5. Obviously, the phosphorylation status of YB‐1 decides on its compartment‐specific functions, as this influences its subcellular location 6, 28. Akt/PKB‐mediated phosphorylation at serine 102 (pYB‐1^S102^) 28 promotes nuclear YB‐1 translocation 6, 36, 37 and affects YB‐1‐dependent gene activity, as demonstrated for the promoter of *epidermal growth factor receptor* 29 as well as for *CCL5* 6 and—as we demonstrate now—for the fourth intron region within the *IL10* gene, an important anti‐inflammatory mediator.

Progression of chronic renal inflammation/kidney diseases critically depends on the decision between resolution and persistence of inflammation. Recently, we demonstrated that *Yb1* ^*+/−*^ mice are protected in a renal inflammation/fibrosis model with persistent a pathological condition, namely unilateral ureteral obstruction (UUO) 24. On the contrary, in I/R, the insult is initial and transient, which allows to analyse not only the onset and the progression of inflammation but also repair mechanisms that are more efficient at shorter ischaemia times. Countervailing effects of YB‐1 were observed during the course of this model and the perception that YB‐1 is involved not only in the onset but also in the resolution of inflammation is supported by the different outcome in WT and *Yb1* ^*+/−*^ mice following I/R. While the expression of markers of inflammation (CCL5) and of fibrosis (Col1A1) was alleviated in the early‐phase of inflammation (days 1 and 5) in *Yb1* ^*+/−*^ mice compared to their WT littermates, they were increased in the later phase (day 21). Thus, factors induced at early time‐points might be responsible for the limited regeneration in the subsequent course of the disease. This applies, for NGAL, a well‐established marker of both renal injury and cell regeneration, which was expressed to a lesser extent at day 5 in the established inflammatory phase after I/R in *Yb1* ^*+/−*^ mice. Furthermore, a key factor for a rapid and effective response to hypoxia includes HIF‐1α that is regulated by YB‐1 at the gene and/or mRNA level 38, 39. HIF‐1α protein expression is enhanced by direct YB‐1 binding to and subsequent activation of the translation of HIF‐1α mRNA 38.

Another important feature to suppress the immune response is the generation of IL‐10. Expression of IL‐10 is achieved by various cells, is of high complexity and tightly regulated. Besides gene expression, additional post‐translational regulators are involved that include for example miR‐21 as demonstrated in macrophages 40. In human Tregs and in murine natural killer (NK) cells, exogenous IL‐2 mediates Stat5 and Stat4 binding to the fourth intron of *IL10* 41, 42. Recently, Hedrich and colleagues demonstrated that the recruitment of Stat3 and Stat5 to the promoter and to the fourth intron of the *IL10* gene in T cells varies following stimulation with different cytokines and that the imbalance between pStat3 and pStat5 in T cells from systemic lupus erythematosus (SLE) patients contributes to an increased IL‐10 expression 21.

In renal I/R, IL‐10 expression is achieved among others by regulatory T cells (Tregs). An adoptive transfer of these cells in renal I/R exerts protective effects that were lost when IL‐10‐deficient Tregs were transferred 17. However, Tregs are not the only cell population that produces IL‐10 in kidneys. Thus, in Treg‐depleted mice, renal IL‐10 expression was still markedly increased in the late renal recovery phase following I/R 15. Along these lines, observations from others 19, 20 and our group proved that mesangial and tubular cells also account for IL‐10 production upon an (inflammatory) insult. The regulation of IL‐10 expression through YB‐1 in both resident renal and immune cells *via* the fourth intron was demonstrated in the present study *via* cell culture assays and two *in vivo* models of renal inflammation*,* namely in LPS‐triggered and sterile inflammation following renal I/R.

In summary, our study provides further evidence for the indispensability of YB‐1 as an adaptor for the immune response and its decisive role for IL‐10 expression. Thus, fine‐tuning of the cellular YB‐1 content and its subcellular localization is crucial in systemic inflammatory processes.

## Conflict of interests

The authors confirm that there are no conflict of interests.

## References

[1] Lasham A , Print CG , Woolley AG , *et al* YB‐1: oncoprotein, prognostic marker and therapeutic target? Biochem J. 2013; 449: 11–23. 23216250 10.1042/BJ20121323

[2] Kohno K , Izumi H , Uchiumi T , *et al* The pleiotropic functions of the Y‐box‐binding protein, YB‐1. BioEssays. 2003; 25: 691–8. 12815724 10.1002/bies.10300

[3] Raffetseder U , Liehn EA , Weber C , *et al* Role of cold shock Y‐box protein‐1 in inflammation, atherosclerosis and organ transplant rejection. Eur J Cell Biol. 2012; 91: 567–75. 21943779 10.1016/j.ejcb.2011.07.001

[4] Hanssen L , Alidousty C , Djudjaj S , *et al* YB‐1 is an early and central mediator of bacterial and sterile inflammation in vivo. J Immunol. 2013; 191: 2604–13. 23872051 10.4049/jimmunol.1300416

[5] Kang S , Lee TA , Ra EA , *et al* Differential control of interleukin‐6 mrna levels by cellular distribution of YB‐1. PLoS ONE. 2014; 9: e112754. 25398005 10.1371/journal.pone.0112754 PMC4232504

[6] Alidousty C , Rauen T , Hanssen L , *et al* Calcineurin‐mediated YB‐1 Dephosphorylation Regulates CCL5 Expression during Monocyte Differentiation. J Biol Chem. 2014; 289: 21401–12. 24947514 10.1074/jbc.M114.562991 PMC4118104

[7] Krohn R , Raffetseder U , Bot I , *et al* Y‐box binding protein‐1 controls CC chemokine ligand‐5 (CCL5) expression in smooth muscle cells and contributes to neointima formation in atherosclerosis‐prone mice. Circulation. 2007; 116: 1812–20. 17893273 10.1161/CIRCULATIONAHA.107.708016

[8] Raffetseder U , Rauen T , Djudjaj S , *et al* Differential regulation of chemokine CCL5 expression in monocytes/macrophages and renal cells by Y‐box protein‐1. Kidney Int. 2009; 75: 185–96. 18800033 10.1038/ki.2008.457

[9] Hesketh EE , Czopek A , Clay M , *et al* Renal ischaemia reperfusion injury: a mouse model of injury and regeneration. J Vis Exp. 2014; (88): 51816. 10.3791/51816 PMC4188040 24961244

[10] Stroo I , Stokman G , Teske GJ , *et al* Chemokine expression in renal ischemia/reperfusion injury is most profound during the reparative phase. Int Immunol. 2010; 22: 433–42. 20410256 10.1093/intimm/dxq025 PMC2877810

[11] Tilney NL , Guttmann RD . Effects of initial ischemia/reperfusion injury on the transplanted kidney. Transplantation. 1997; 64: 945–7. 9381538 10.1097/00007890-199710150-00001

[12] Devarajan P . Update on mechanisms of ischemic acute kidney injury. J Am Soc Nephrol. 2006; 17: 1503–20. 16707563 10.1681/ASN.2006010017

[13] Star RA . Treatment of acute renal failure. Kidney Int. 1998; 54: 1817–31. 9853246 10.1046/j.1523-1755.1998.00210.x

[14] Mehta RL , Pascual MT , Soroko S , *et al* ; Program to Improve Care in Acute Renal D . Spectrum of acute renal failure in the intensive care unit: the PICARD experience. Kidney Int. 2004; 66: 1613–21. 15458458 10.1111/j.1523-1755.2004.00927.x

[15] Gandolfo MT , Jang HR , Bagnasco SM , *et al* Foxp3 + regulatory T cells participate in repair of ischemic acute kidney injury. Kidney Int. 2009; 76: 717–29. 19625990 10.1038/ki.2009.259

[16] Li L , Huang L , Ye H , *et al* Dendritic cells tolerized with adenosine A(2)AR agonist attenuate acute kidney injury. J Clin Invest. 2012; 122: 3931–42. 23093781 10.1172/JCI63170 PMC3484444

[17] Kinsey GR , Sharma R , Huang L , *et al* Regulatory T cells suppress innate immunity in kidney ischemia‐reperfusion injury. J Am Soc Nephrol. 2009; 20: 1744–53. 19497969 10.1681/ASN.2008111160 PMC2723989

[18] Ostmann A , Paust HJ , Panzer U , *et al* Regulatory T cell‐derived IL‐10 ameliorates crescentic GN. J Am Soc Nephrol. 2013; 24: 930–42. 23641052 10.1681/ASN.2012070684 PMC3665390

[19] Sinuani I , Averbukh Z , Gitelman I , *et al* Mesangial cells initiate compensatory renal tubular hypertrophy via IL‐10‐induced TGF‐beta secretion: effect of the immunomodulator AS101 on this process. Am J Physiol Renal Physiol. 2006; 291: F384–94. 16571592 10.1152/ajprenal.00418.2005

[20] Dhande I , Ali Q , Hussain T . Proximal tubule angiotensin AT2 receptors mediate an anti‐inflammatory response via interleukin‐10: role in renoprotection in obese rats. Hypertension. 2013; 61: 1218–26. 23547236 10.1161/HYPERTENSIONAHA.111.00422 PMC3709839

[21] Hedrich CM , Rauen T , Apostolidis SA , *et al* Stat3 promotes IL‐10 expression in lupus T cells through trans‐activation and chromatin remodeling. Proc Natl Acad Sci U S A. 2014; 111: 13457–62. 25187566 10.1073/pnas.1408023111 PMC4169908

[22] Rauen T , Raffetseder U , Frye BC , *et al* YB‐1 acts as a ligand for Notch‐3 receptors and modulates receptor activation. J Biol Chem. 2009; 284: 26928–40. 19640841 10.1074/jbc.M109.046599 PMC2785380

[23] Lu ZH , Books JT , Ley TJ . YB‐1 is important for late‐stage embryonic development, optimal cellular stress responses, and the prevention of premature senescence. Mol Cell Biol. 2005; 25: 4625–37. 15899865 10.1128/MCB.25.11.4625-4637.2005 PMC1140647

[24] Wang J , Gibbert L , Djudjaj S , *et al* Therapeutic nuclear shuttling of YB‐1 reduces renal damage and fibrosis. Kidney Int. 2016; 90: 1226–37. 27591085 10.1016/j.kint.2016.07.008

[25] Djudjaj S , Chatziantoniou C , Raffetseder U , *et al* Notch‐3 receptor activation drives inflammation and fibrosis following tubulointerstitial kidney injury. J Pathol. 2012; 228: 286–99. 22806125 10.1002/path.4076

[26] Mertens PR , Harendza S , Pollock AS , *et al* Glomerular mesangial cell‐specific transactivation of matrix metalloproteinase 2 transcription is mediated by YB‐1. J Biol Chem. 1997; 272: 22905–12. 9278454 10.1074/jbc.272.36.22905

[27] En‐Nia A , Yilmaz E , Klinge U , *et al* Transcription factor YB‐1 mediates DNA polymerase alpha gene expression. J Biol Chem. 2005; 280: 7702–11. 15615704 10.1074/jbc.M413353200

[28] Sutherland BW , Kucab J , Wu J , *et al* Akt phosphorylates the Y‐box binding protein 1 at Ser102 located in the cold shock domain and affects the anchorage‐independent growth of breast cancer cells. Oncogene. 2005; 24: 4281–92. 15806160 10.1038/sj.onc.1208590

[29] Stratford AL , Habibi G , Astanehe A , *et al* Epidermal growth factor receptor (EGFR) is transcriptionally induced by the Y‐box binding protein‐1 (YB‐1) and can be inhibited with Iressa in basal‐like breast cancer, providing a potential target for therapy. Breast Cancer Res. 2007; 9: R61 17875215 10.1186/bcr1767 PMC2242657

[30] Raffetseder U , Rauen T , Boor P , *et al* Extracellular YB‐1 blockade in experimental nephritis upregulates Notch‐3 receptor expression and signaling. Nephron Exp Nephrol. 2011; 118: e100–8. 21372592 10.1159/000324209

[31] Soranno DE , Rodell CB , Altmann C , *et al* Delivery of interleukin‐10 via injectable hydrogels improves renal outcomes and reduces systemic inflammation following ischemic acute kidney injury in mice. Am J Physiol Renal Physiol. 2016; 311: 362–72. 10.1152/ajprenal.00579.2015 PMC5008670 26962109

[32] Sorrenti V , Di Giacomo C , Acquaviva R , *et al* Toxicity of ochratoxin a and its modulation by antioxidants: a review. Toxins. 2013; 5: 1742–66. 24152986 10.3390/toxins5101742 PMC3813909

[33] Hanssen L , Frye BC , Ostendorf T , *et al* Y‐box binding protein‐1 mediates profibrotic effects of calcineurin inhibitors in the kidney. J Immunol. 2011; 187: 298–308. 21606250 10.4049/jimmunol.1100382

[34] van Roeyen CR , Eitner F , Martinkus S , *et al* Y‐box protein 1 mediates PDGF‐B effects in mesangioproliferative glomerular disease. J Am Soc Nephrol. 2005; 16: 2985–96. 16093451 10.1681/ASN.2004111009

[35] Dong W , Wang H , Shahzad K , *et al* Activated protein C ameliorates renal ischemia‐reperfusion injury by restricting y‐box binding protein‐1 ubiquitination. J Am Soc Nephrol. 2015; 26: 2789–99. 26015455 10.1681/ASN.2014080846 PMC4625674

[36] Sinnberg T , Sauer B , Holm P , *et al* MAPK and PI3K/AKT mediated YB‐1 activation promotes melanoma cell proliferation which is counteracted by an autoregulatory loop. Exp Dermatol. 2012; 21: 265–70. 22417301 10.1111/j.1600-0625.2012.01448.x

[37] Wu J , Lee C , Yokom D , *et al* Disruption of the Y‐box binding protein‐1 results in suppression of the epidermal growth factor receptor and HER‐2. Cancer Res. 2006; 66: 4872–9. 16651443 10.1158/0008-5472.CAN-05-3561

[38] El‐Naggar AM , Veinotte CJ , Cheng H , *et al* Translational Activation of HIF1alpha by YB‐1 Promotes Sarcoma Metastasis. Cancer Cell. 2015; 27: 682–97. 25965573 10.1016/j.ccell.2015.04.003

[39] Rauen T , Frye BC , Wang J , *et al* Cold shock protein YB‐1 is involved in hypoxia‐dependent gene transcription. Biochem Biophys Res Commun. 2016; 478: 982–7. 27524241 10.1016/j.bbrc.2016.08.064

[40] Merline R , Moreth K , Beckmann J , *et al* Signaling by the matrix proteoglycan decorin controls inflammation and cancer through PDCD4 and MicroRNA‐21. Sci Signal. 2011; 4: ra75 22087031 10.1126/scisignal.2001868 PMC5029092

[41] Tsuji‐Takayama K , Suzuki M , Yamamoto M , *et al* The production of IL‐10 by human regulatory T cells is enhanced by IL‐2 through a STAT5‐responsive intronic enhancer in the IL‐10 locus. J Immunol. 2008; 181: 3897–905. 18768844 10.4049/jimmunol.181.6.3897

[42] Grant LR , Yao ZJ , Hedrich CM , *et al* Stat4‐dependent, T‐bet‐independent regulation of IL‐10 in NK cells. Genes Immun. 2008; 9: 316–27. 18401353 10.1038/gene.2008.20 PMC2689787

